# Electroacupuncture at Zusanli regulates the pathological phenotype of inflammatory bowel disease by modulating the NLRP3 inflammasome pathway

**DOI:** 10.1002/iid3.1366

**Published:** 2024-08-09

**Authors:** Yanqiang Chen, Miaomiao Cai, Boyuan Shen, Changchang Fan, Xiang Zhou

**Affiliations:** ^1^ Hubei Provincial Hospital of Integrated Chinese and Western Medicine Wuhan Hubei China; ^2^ College of Life Science and Health Wuhan University of Science and Technology Wuhan Hubei China

**Keywords:** electroacupuncture, IBD, NLRP3, Zusanli

## Abstract

**Background:**

This study sought to explore the effect of electroacupuncture (EA) intervention at Zusanli (ST36) acupoint on modulating the NLRP3 inflammasome pathway for treating inflammatory bowel disease (IBD).

**Methods:**

C57BL/6 mice were administrated with 3% dextran sulfate sodium (DSS) to construct the IBD model. DSS mice were then administrated with EA (10 Hz, 1.5 mA) at ST36 for 7 days or intragastric administration of sulfasalazine (SASP) each day during the entire course. The control group animals were administered with distilled water. Then, partial least squares discriminant analysis revealed differences in the relative content of metabolites. The pathological changes of colon and spleen tissues were observed by H&E and immunohistochemistry (IHC) staining. qPCR determined the mRNA expression levels, while ELISA and western blot analysis determined the protein expression.

**Results:**

Compared with the control groups, DSS‐induced decreases of body weight were reversed after EA stimulation at ST36 or SASP treatment. The DAI of DSS mice was significantly higher relative to the control groups, whereas the DAI of DSS mice were decreased after EA stimulation at ST36 or SASP treatment. The intestinal weight/length ratio increased significantly in DSS groups; however, EA at ST36 significantly improved the macroscopic/microscopic characteristics and the weight and length of the colon. EA reversed inflammation and leukocyte infiltration and normalized the elevated levels of IL‐1β, IL‐18, and NLRP3. Furthermore, EA improved the expression levels of ZO‐1, occludin, and claudin 1, exhibiting normalization of the colon's tight junctions.

**Conclusions:**

EA at Zusanli acupoint of colon tissue significantly improved the pathological phenotype, showing a therapeutic effect on IBD.

## Graphical abstract

Schematics of electroacupuncture (EA) at “Zusanli” (ST36) effects on dextran sulfate sodium (DSS)‐induced acute colitis: EA at ST36 in inflammatory bowel disease (IBD) model decreases the expression of BAX and BAK1, inhibits the formation of BAX/BAK1 pore and prevents mtDNA leakage from colitis, which results in the inhibition of NLRP3 inflammasomes and abating the expression and splicing of GSDMD, IL‐1β, and IL‐18. Meanwhile, the release of proinflammatory factor IL‐4/IL‐6/IL‐8 and anti‐inflammatory factor IL‐10 were reversed after EA, which is responsible for the decreased cell death. Under the synergistic action of these two aspects, the increase of tight‐junction protein and the reduction of key proteins of tryptophan metabolism such as IDO and KMO regulate cell death caused by inflammation, repair of damaged tissues, and attenuation of colitis.

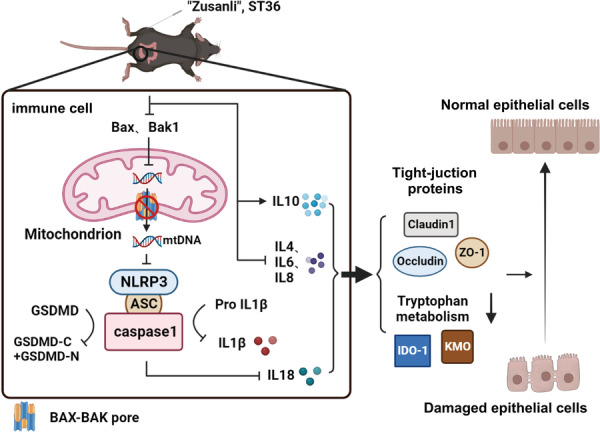

## 1 INTRODUCTION

Inflammatory bowel disease (IBD), which includes ulcerative colitis (UC) and Crohn's disease (CD), is a chronic and recurring inflammatory disease primarily affecting the gastrointestinal tract.^1^, ^2^ The main symptoms include severe diarrhea, abdominal pain, electrolyte loss, and bleeding.^3^ Prior studies suggest that the core pathology of IBD stems from an autoimmune inflammatory process, leading to the imbalance in release of proinflammatory and anti‐inflammatory cytokines and gut microbiota metabolites. This activation culminates in a substantial production of reactive oxygen species, precipitating oxidative damage to intestinal epithelial cells.^4^, ^5^ As reported by the Chinese Institute for Disease Control and Prevention, there were approximately 350,000 cases of IBD from 2005 to 2014, and the incidence of IBD was 1.74 (95% CI: 1.08) per 100,000.^6^ IBD is susceptible to recurrence, and no effective treatment or technique provides long‐term remission for the majority of patient.^7^

There are several primary therapeutic approaches for IBD, such as sulfasalazine (SASP), steroids, immunosuppressive agents, and new biological agents. However, long‐term treatment with steroids and immunosuppressants can cause serious adverse reactions.^8^ The clinical introduction of tumor necrosis factor (TNF) inhibitors are the common strategy for the treatment of patient with IBD and had impressive efficacy to achieve near‐remission and long‐term improvement in function and quality of life and to alter the natural history of CD and UC.^9^ However, the current anti‐TNF therapy is not fully effective in all patients with IBD, and approximately 40% of patients with anti‐TNF treatment will suffer from primary failure in clinical trials,^10^ which calls for new therapeutic strategies.

As a traditional Chinese medicine (TCM), electroacupuncture (EA) has been widely used in treating in chronic and acute diseases.^11^, ^12^ EA has been approved by the US Food and Drug Administration and the World Health Organization.^13^ Since the 1990s, there have been increasing clinical studies on acupuncture treatment for IBD. The current study demonstrated that EA can effectively control IBD by multitargeted regulation of the body's physiological balance.^14^, ^15^ Previous studies have shown that EA modulates T‐cell‐mediated immunity, activates natural killer (NK) cells and macrophages, and prompts cytokine release.^16^, ^17^ Increasing evidence have supported the abnormal activation of the NLRP3 inflammasome in dextran sulfate sodium (DSS)‐colitis and TNBS‐colitis.^18^, ^19^ The main downstream comments of NLRP3 inflammasome are IL‐1β and IL‐18. IL‐1β is a known inflammatory cytokine and is involved in local and systemic responses to injury, infection, and inflammation.^20^, ^21^ The abnormal levels of IL‐18 are related to intestinal inflammation in IBD.^22^, ^23^ Also, EA stimulation can alleviate irritable bowel syndrome by regulating IL‐18 and gut microbial dysbiosis in a trinitrobenzene sulfonic acid‐induced post‐inflammatory animal model, supporting a potential positive effect of EA in visceral hypersensitivity and gastrointestinal tract.^24^ Furthermore, clinical evidence has shown that sacral nerve stimulation therapy with EA may exert anti‐inflammatory efficacy in patients with UC.^25^ Furthermore, clinical evidence supports EA's efficacy in mitigating the progression of inflammation‐related diseases, such as rheumatoid arthritis, osteoarthritis, and asthma.^26^, ^27^, ^28^ Previous studies have shown that using EA on the Zusanli acupoint can reduce IBD severity ^14^, ^15^

This investigation seeks to assess the influence of EA at the Zusanli acupoint on the pathophysiology of IBD. We hypothesize that EA attenuates NLRP3 inflammasome activation, engages the efferent fibers of the vagus nerve, initiates the cholinergic anti‐inflammatory pathway, conveys signals to intestinal neurons, suppresses IL‐1β/IL‐18 secretion, and ultimately downregulates tryptophan metabolism, contributing to the attenuation of IBD.

## 2 MATERIALS AND METHODS

### 2.1 Construction of IBD animal model

The animal experiment design followed the “3R” principle. The mice were kept at the Wuhan University of Science and Technology animal center in individually ventilated cages with a 12 light–dark cycle environment at 25 ± 1°C. The mice had free access to food and water. All animal research in this study were reported in accordance with ARRIVE guidelines. The Institutional Animal Care and Use Committee of Wuhan University of Science and Technology approved the animal experiments, and the Guide for the Use of Laboratory Animals (National Academy Press) was followed. All mice were fasted for 12 h and were in a state of fecal emptying. Acute colitis was induced with 3% (wt/vol) DSS (molecular weight, 36−50 kDa; Yeason) dissolved in drinking water and was administered for 7 days. To prevent the degradation of DSS from affecting the accuracy of experimental results, DSS was changed every third day. Body weight and fecal status were recorded daily. The successful establishment of acute colitis model was based on the following criteria: (1) Weight loss, weakened vitality, diarrhea, hematochezia, and other symptoms; (2) Colonic congestion, edema, shortening, varying degrees of inflammatory cell infiltration, and epithelial cell damage.

### 2.2 Experimental animal design

**Figure 1:**
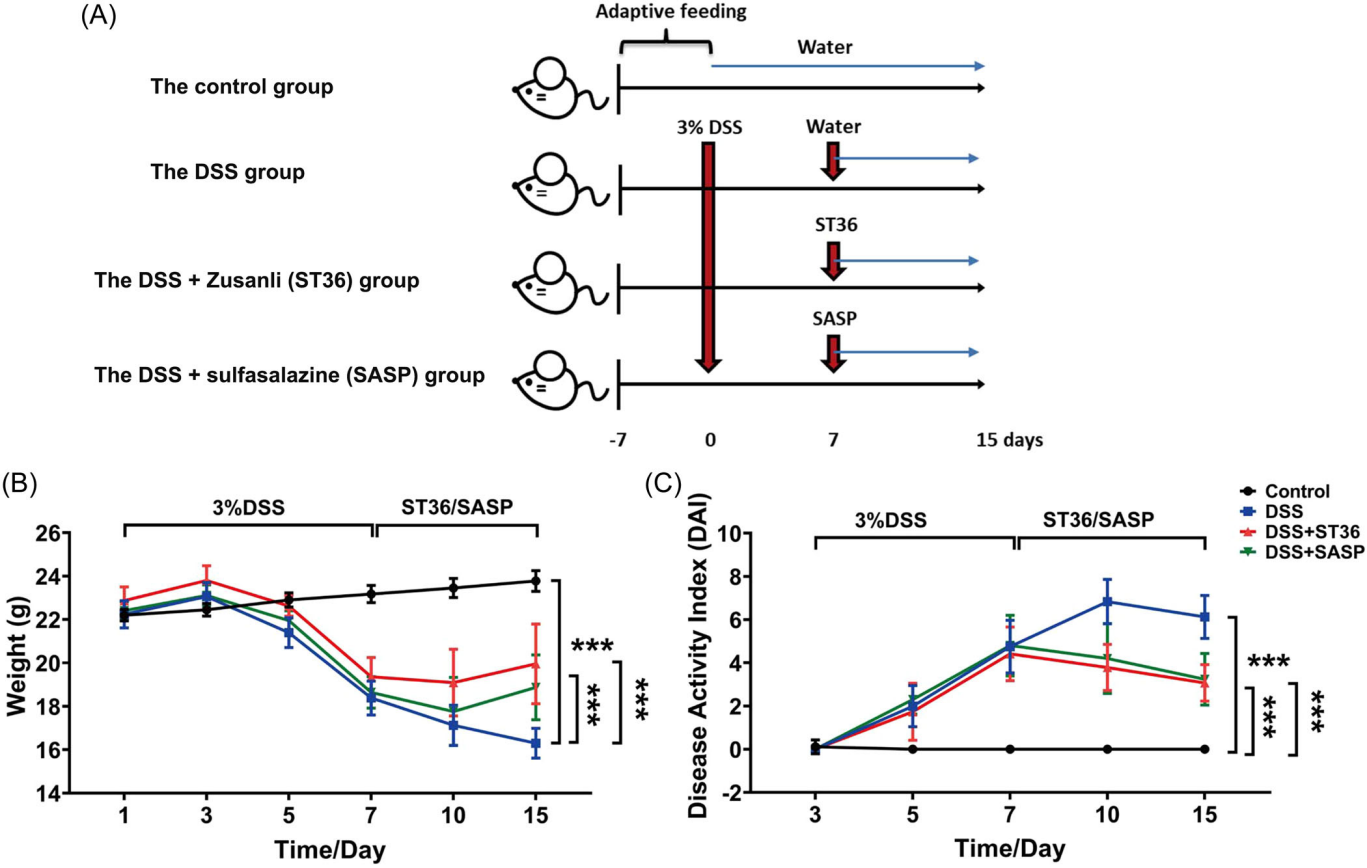
Electroacupuncture ameliorates DSS‐induced colitis in C57BL/6 mice. Mice were administered with 3% (wt/vol) (DSS for 7 days and/or various frequencies of EA for 30 min daily. Seven days later, mice were killed and examined for colitis by macroscopic lesions and microscopic sections. (A) Establishment of IBD disease model and acupuncture protocol. (B) The IBD mice constructed by DSS were administrated with EA stimulation on ST36 and SASP. Then, the body weight of mice was determined. (C) Effects of EA and SASP on DAI of mice. ***p < .001. DSS, dextran sulfate sodium; EA, electroacupuncture; IBD, inflammatory bowel disease; SASP, sulfasalazine.

A total of 40 C57BL/6 mice (6−8 weeks old) were randomly divided into four groups, with 10 mice (male: 20−22 g) in each group, as illustrated in Figure 1A. Mice in the DSS groups received the 3% (wt/vol) DSS dissolved in drinking water for 7 days. The control group animals were administered with distilled water. For DSS and bilateral Zusanli (ST36) groups, DSS mice were anesthetized with isoflurane, and EA needles were subcutaneously inserted into ST36, which have similar characteristics to those in humans. During the process of anesthetization, a gas anesthesia device integrated with a small animal in vivo imaging system was used. The initial concentration of anesthetic isoflurane was set at 2%. After the anesthetic filled the induction chamber for 1 min, mice were placed inside. The induction chamber was then closed, and the mice were allowed to become fully anesthetized, a process that typically took about 2−3 min. Subsequently, the maintenance concentration was adjusted to 0.5%−1%. The anesthetic concentration was dynamically adjusted based on the condition of the mouse, ensuring that even mice experiencing significant weight loss can tolerate the procedure. After that, the needles were connected to a Trio 300 electrical stimulator (Grand Medical Instrument Co., Ltd) with an electric stimulation (10 Hz). The intensity of electrical stimulation was sufficient to cause visible muscle twitching. This examination was performed for 30 min each day for 7 consecutive days; finally, the mice were killed immediately after the final EA under 3%−5% isoflurane anesthesia, and the mouse intestines were collected for metabolomic analyses. In DSS and SASP groups, DSS mice received intragastric administration of SASP each day during the entire course. The four groups of mice were treated for 7 days, and clinical indicators were observed and recorded, including weight loss, stool consistency, and rectal bleeding, after mice were killed using cervical dislocation immediately after the final EA under 3%−5% isoflurane anesthesia.

### 2.3 Detection of histopathological changes

To test whether acupuncture at Zusanli can help alleviate the pathological symptoms of IBD model mice, colon and spleen tissues were collected. Then, tissues were fixed in 4% paraformaldehyde for 24 h, paraffin‐embedded, and sectioned into 5 μm serial sections. For hematoxylin and eosin (H&E) staining, H&E were used successively. For immunohistochemistry (IHC) staining, the slices were dewaxed and immersed in the antigen‐repairing solution for 10 min and then sealed with sealer after they returned to room temperature. Primary antibody was used for immunohistochemical staining. The goat anti‐rabbit IgG secondary antibodies (Servicebio; G1216‐200T) were used at an appropriate concentration (1:1000). The 3,3ʹ‐diaminobenzidine (DAB) solution (Servicebio; G1216‐200T) was used for color development. Finally, the nuclei were counterstained with hematoxylin. Intestinal histological evaluation was carried out to assess the disease severity using the tissue sample.

### 2.4 ELISA, qPCR, and western blot analysis

After the last stimulation with EA, the mice were killed (3%−5% isoflurane). Colon tissues were cut into pieces were immediately stored at −80°C liquid nitrogen. For ELISA assay, the levels of IL‐10 L‐18, and IL‐1β in tissue samples were determined using the commercial ELISA kits.

For qPCR analysis, total RNA was extracted using TRIZOL from different groups according to the manufacturer's instructions for the determination of IL‐4, IL‐6, IL‐18, IL‐10, IL‐1β, and NLRP3 expression levels. The primer sequences used in this study are reported in Table 1. RNA quality was assessed based on the A260/A280 ratio. Total RNA (1.5 μg) was used to generate first‐strand cDNA using SuperScript III Reverse Transcriptase (1 μL). The RT‐PCR reaction mixtures were prepared using a SuperArray PCR Master Mix (Cat. No. PA‐112). The cDNA was added to each well of the plate and the RT‐PCR was initiated. The cycle conditions were: 95°C for 10 min, 60°C for 15 s, and 60°C for 1 min (40 cycles).

**Table 1:** PCR primer sequences.

Gene name	Forward primer	Reverse primer
IL‐4	GGTCTCAACCCCCAGCTAGT	GCCGATGATCTCTCTCAAGTGAT
IL‐6	TAGTCCTTCCTACCCCAATTTCC	TTGGTCCTTAGCCACTCCTTC
IL‐8	CAAGGCTGGTCCATGCTCC	TGCTATCACTTCCTTTCTGTTGC
IL‐10	GCTCTTACTGACTGGCATGAG	CGCAGCTCTAGGAGCATGTG
IL‐1β	GCAACTGTTCCTGAACTCAACT	ATCTTTTGGGGTCCGTCAACT
NLRP3	ATTACCCGCCCGAGAAAGG	TCGCAGCAAAGATCCACACAG

The Bax, Bak1, NLRP3, IL‐1β, and GSDMD‐N expression levels were detected by western blot analysis. After treatment with EA, the mice were killed followed by an incision of the abdominal skin and peritoneum. The colon tissue was collected and cut into small pieces and immediately stored at −80°C in liquid nitrogen. Briefly, the homogenates (30 μg) of the colon tissue were separated by sodium dodecyl sulfate‐polyacrylamide gel electrophoresis (SDS‐PAGE) and then transferred to nitrocellulose membranes. The proteins were blocked with Tris‐buffered saline (TBST)/5% milk, followed by incubation with polyclonal antibodies at 4°C overnight. After washing, the membranes were incubated with 1:2000 diluted secondary peroxidase‐conjugated antibodies in TBST with 0.01% Tween 20. The ECL was used to detect the proteins according to the manufacturer's instructions.

### 2.5 Statistical analysis

Statistical analyses were performed using the GraphPad Prism (Version 9). Comparisons among multiple groups were analyzed by analysis of variance (ANOVA) with Tukey's Honest Significant Difference (HSD) test. *p* < .05 was set as the threshold indicating statistical significance for all above analyses.

## 3 RESULTS

### 3.1 The disease progression of colitis is delayed after EA stimulation at ST36

Compared to the control group, the body weight of mice treated with DSS significantly decreased (Figure 1B). Notably, the decreases of body weight in DSS mice were reversed after EA stimulation at ST36. Concomitantly, loss of body weight in DSS groups was also offset following SASP treatment (Figure 1B). Meanwhile, DSS treatment increased DAI relative to the control mice; however, the elevation of DAI in DSS groups was reduced after EA stimulation at ST36 or SASP administration (Figure 1C). The determination of DAI score is in Table 2.

**Table 2:** Determination of DAI score.

Score	Weight loss (%)	Stool consistency	Intestinal bleeding
0	0	Normal	Negative hemocult
1	1−5	Soft stool	
2	5−10	Mucoid stool	Macroscopic bloody stool
3	10−20	Dilute liquid stool	
4	>20	——	Gross rectal bleeding

*Note*: The DAI was scored from three aspects: weight loss, stool consistency, and degree of intestinal bleeding, and the sum of the three indicators was DAI score.

### 3.2 EA stimulation at ST36 restores colon length and spleen size in acute colitis model mice

**Figure 2:**
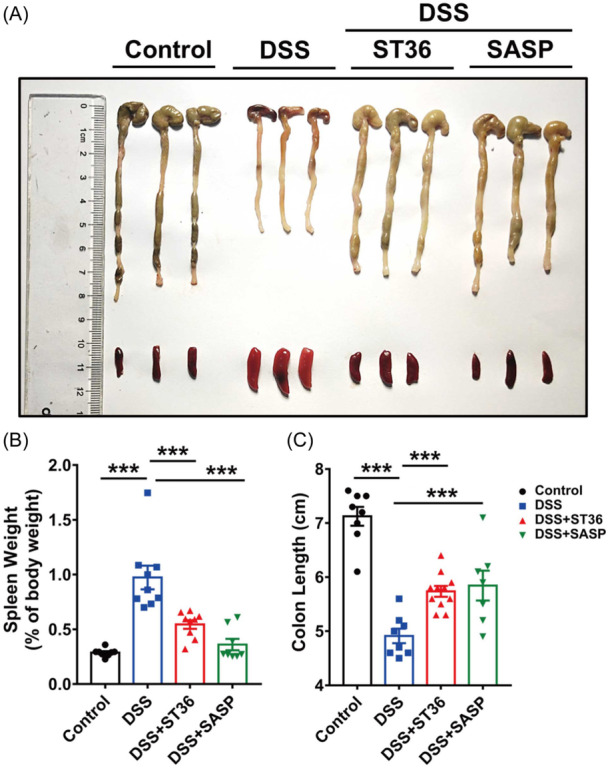
Effects of EA on DSS‐induced colitis in mice. (A) Representative colon and spleen images on Day 15. (B, C) Representative colon lengths and spleen weight of body weight were measured on Day 15. ***p < .001. DSS, dextran sulfate sodium; EA, electroacupuncture; SASP, sulfasalazine.

Previous studies have demonstrated that intrarectal administration with DSS in mice induces colitis resembling IBD in humans.^29^, ^30^ Previous studies have also demonstrated that intrarectal treatment of 3% DSS causes severe macroscopic and microscopic damage in colons.^31^, ^32^, ^33^, ^34^ Therefore, the efficacy of EA on DSS‐induced colitis was evaluated by applying EA at ST36 acupoints. The control group showed no sign or a very low level of macroscopic and microscopic lesions in the colon (Figure 2A). However, macroscopic and microscopic examinations of colons after DSS induction showed inflammation and leukocyte infiltration. In contrast, EA at ST36 acupoints and SASP significantly improved the macroscopic and microscopic features. Moreover, colonic length was decreased and spleen/body weight ratio was increased in DSS‐induced colitis groups relative to the control groups (Figure 2B,C). Notably, EA stimulation at ST36 or SASP both reversed the effects of DSS on the decrease of colonic length and an increase of spleen/body weight ratio. These findings suggested that EA at ST36 acupoints and SASP ameliorated the progression of colitis by significant normalization of intestine and spleen.

### 3.3 EA stimulation at ST36 attenuates epithelial injury and spleen inflammation caused by colitis

**Figure 3:**
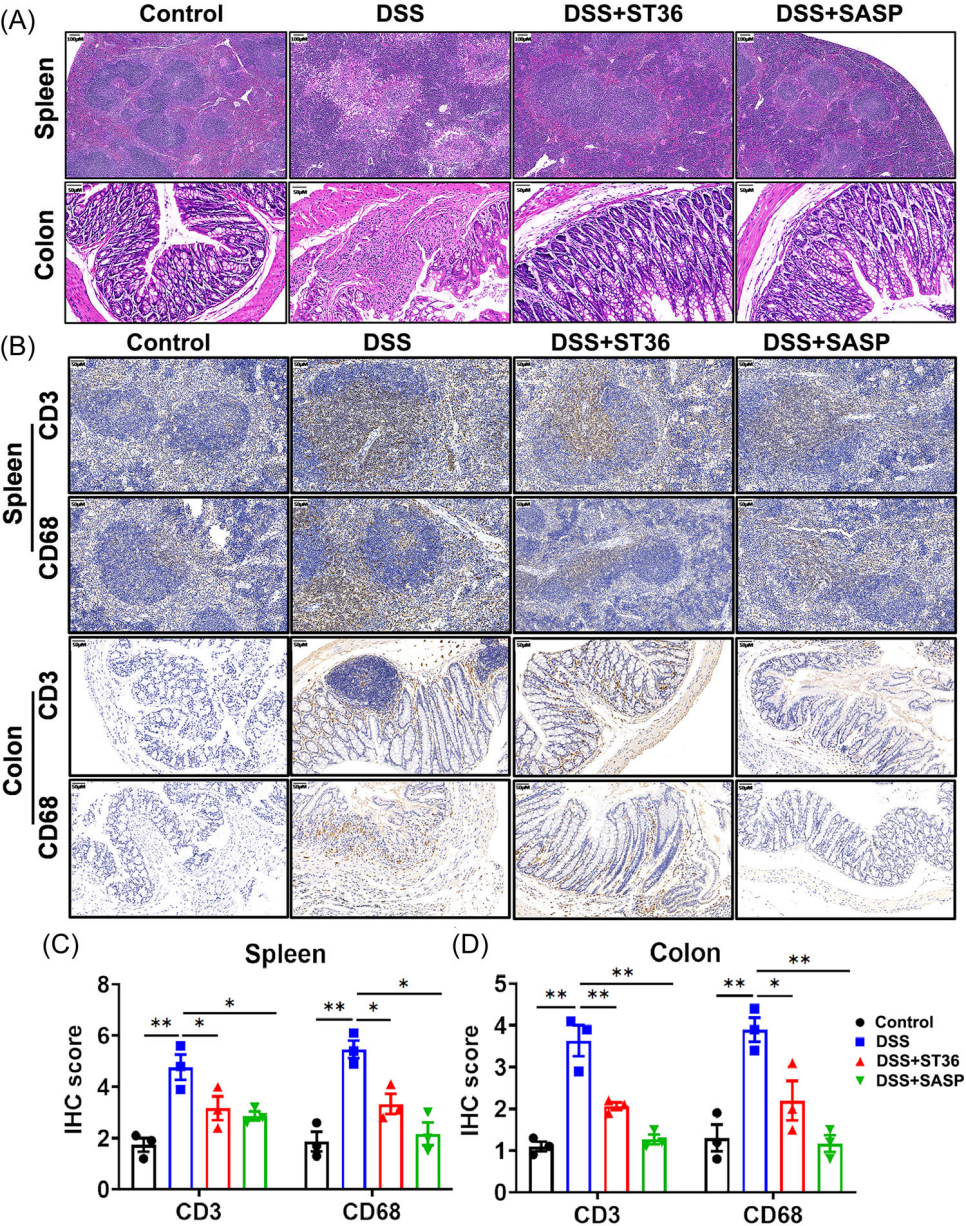
H&E and immunohistochemical (IHC) staining of colon and spleen sections, scale bar, 50 or 100 μm. Mice were administered with 3% (wt/vol) DSS to construct the IBD model. Seven days later, mice were killed and examined for colitis by macroscopic lesions and microscopic sections. (A) Representative H&E image of distal colon sections on Day 15 (*n* = 10). (B) Expression of CD3 and CD68 in colon and spleen tissue of different groups by IHC. (C, D) IHC score of CD3/CD68 in spleen and colon. *p < .05, **p < .01. DSS, dextran sulfate sodium; H&E, hematoxylin and eosin; IBD, inflammatory bowel disease; SASP, sulfasalazine.

The control group showed no sign or a very low level of macroscopic and microscopic lesions in the spleen and colon (Figure 3A). However, there were obvious inflammation and leukocyte infiltration in colon tissues from DSS mice (Figure 3A). In contrast, EA at ST36 acupoints and SASP significantly improved the microscopic features of DSS‐induced colitis (Figure 3A). Interestingly, EA at ST36 acupoints and SASP significantly reduced the increased expression levels of CD3 and CD68 in spleen and colon relative to the DSS groups (Figure 3B−D), indicating that EA and SASP could attenuate the histopathological condition of IBD mice.

### 3.4 Recover of colon's tight junctions in DSS‐induced colitis by electrical stimulation at ST36

**Figure 4:**
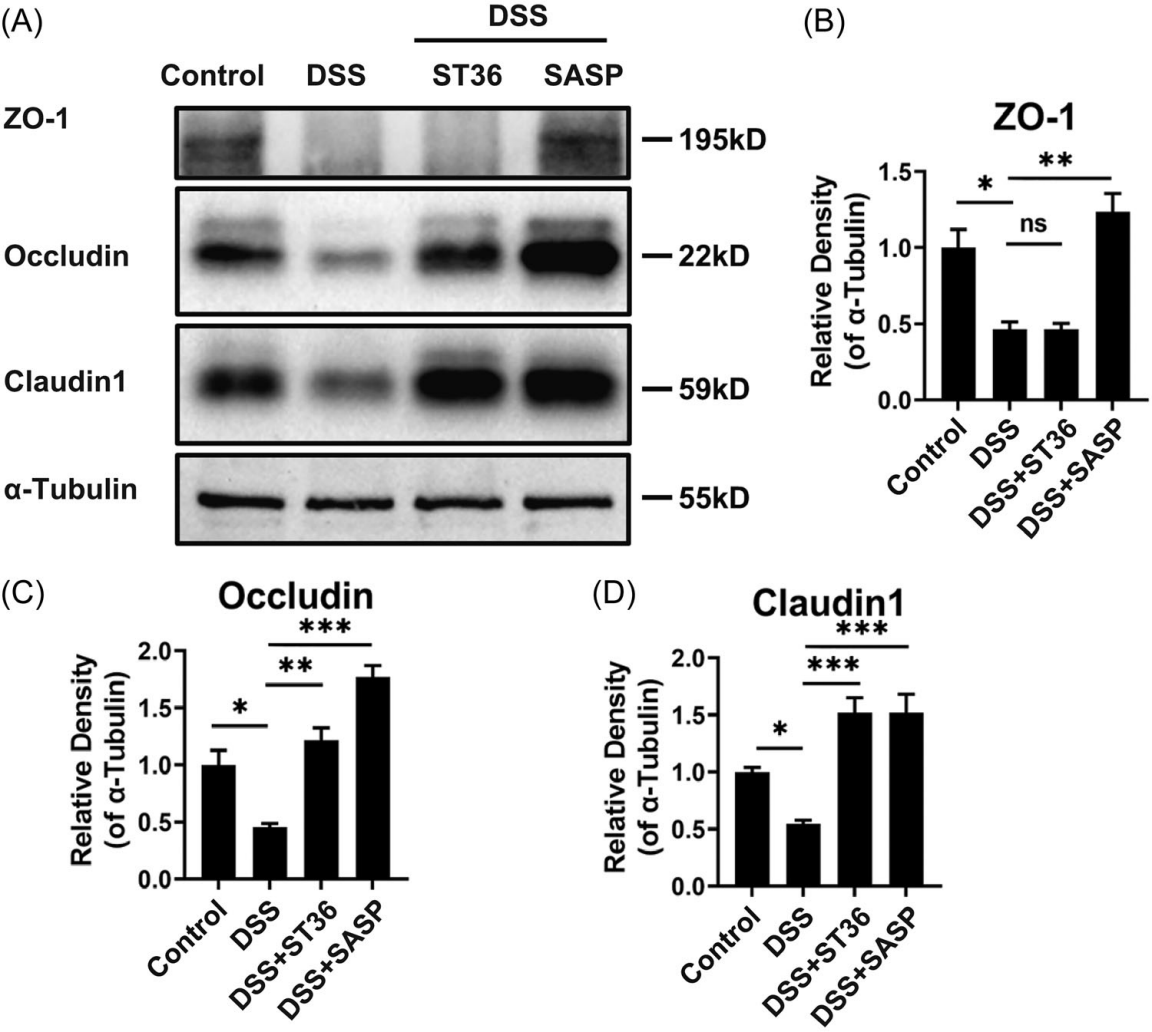
EA at ST36 affects protein expression of colonic compact junction in IBD mice. Mice under DSS conditions were treated with EA at ST36 and SASP. (A) Then, the protein expression of ZO‐1, occludin, and claudin‐1 on IBD mice was detected by western blot analysis assay. (B−D) The binding bands were quantified by the ImageJ software. *p < .05, **p < .01, ***p < .001. DSS, dextran sulfate sodium; EA, electroacupuncture; IBD, inflammatory bowel disease; SASP, sulfasalazine.

Studies have shown a decrease in key tight junction proteins such as ZO‐1 and occludin in IBD and experimental animal models of inflammation.^35^ Compared to the control groups, the obvious decreases of ZO‐1, occluding, and claudin‐1 were detected in the DSS groups, suggesting that DSS in intestinal epithelial cells can lead to the induction of perforation of colon (Figure 4A−D). However, EA at ST36 acupoints had little effect on ZO‐1 expression in DSS mice; whilst, SASP treatment elevated ZO‐1 expression in DSS mice (Figure 4B). Moreover, relative to the DSS groups, EA at ST36 or SASP administration upregulated the expression of occludin and claudin 1 in colonic tissues, indicating the complete normalization of tight junctions in colon. These findings suggest the involvement of occludin and claudin 1 in EA‐mediated alleviation of DSS‐induced colitis.

### 3.5 EA stimulation of ST36 alleviates colonic inflammation in IBD mice

**Figure 5:**
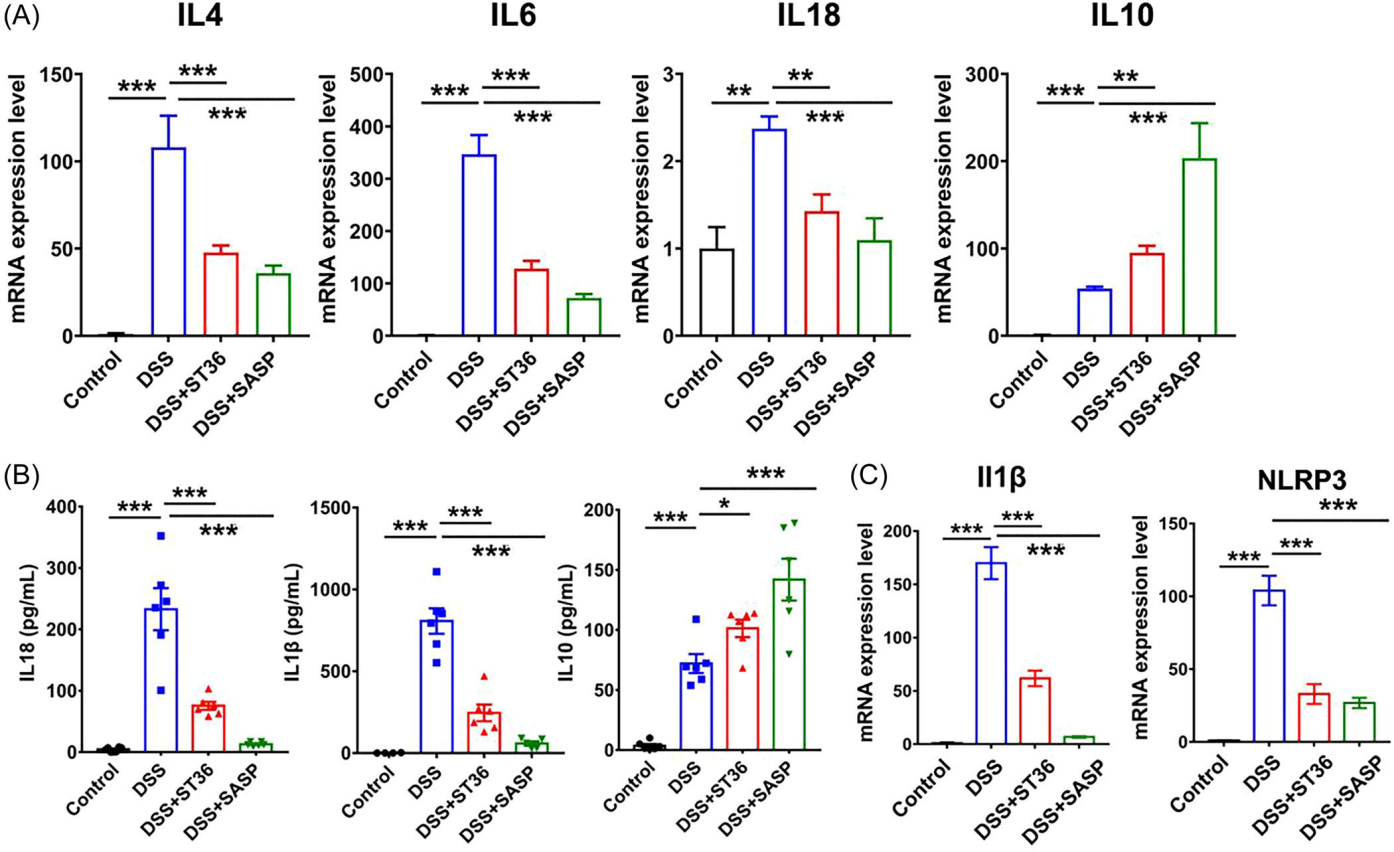
Effect of EA and SASP on expression of proinflammatory cytokines in EA and SASP‐treated mice following DSS exposure. (A) The mRNA levels of IL‐4, IL‐6, and IL‐10 were detected by qPCR. (B) Concentrations of IL‐18, IL‐1β, and IL‐10 in colonic tissues were determined by ELISA assay. (C) The transcripts of IL‐1β and NLRP3 in IBD mice were analyzed by qPCR. *p < .05, **p < .01, ***p < .001. DSS, dextran sulfate sodium; EA, electroacupuncture; IBD, inflammatory bowel disease; SASP, sulfasalazine.

Compared with the control groups, the increased expression of cytokines was observed after DSS treatment, including IL‐4, IL‐6, IL‐8, and IL‐10 (Figure 5A), suggesting that cytokines are involved in the pathogenesis of DSS‐induced colitis.^45^ After the treatment with EA and SASP, we observed significant downregulation of IL‐4, IL‐6, IL‐8 and upregulation of IL‐10 (Figure 5A). Similarly, the enhanced production of IL‐10, IL‐18, IL‐1β (Figure 5B), and mRNA expression of NLRP3 and IL‐1β (Figure 5C) were determined in the colon after DSS treatment. Furthermore, the enhanced transcripts of IL‐1β and NLRP3 in DSS‐treated colons were attenuated after EA at ST36 and SASP treatment. These findings suggest EA at ST36 may alleviate colonic inflammation in the progression of IBD.

### 3.6 EA stimulation at ST36 inhibited the activation levels of NLRP3

**Figure 6:**
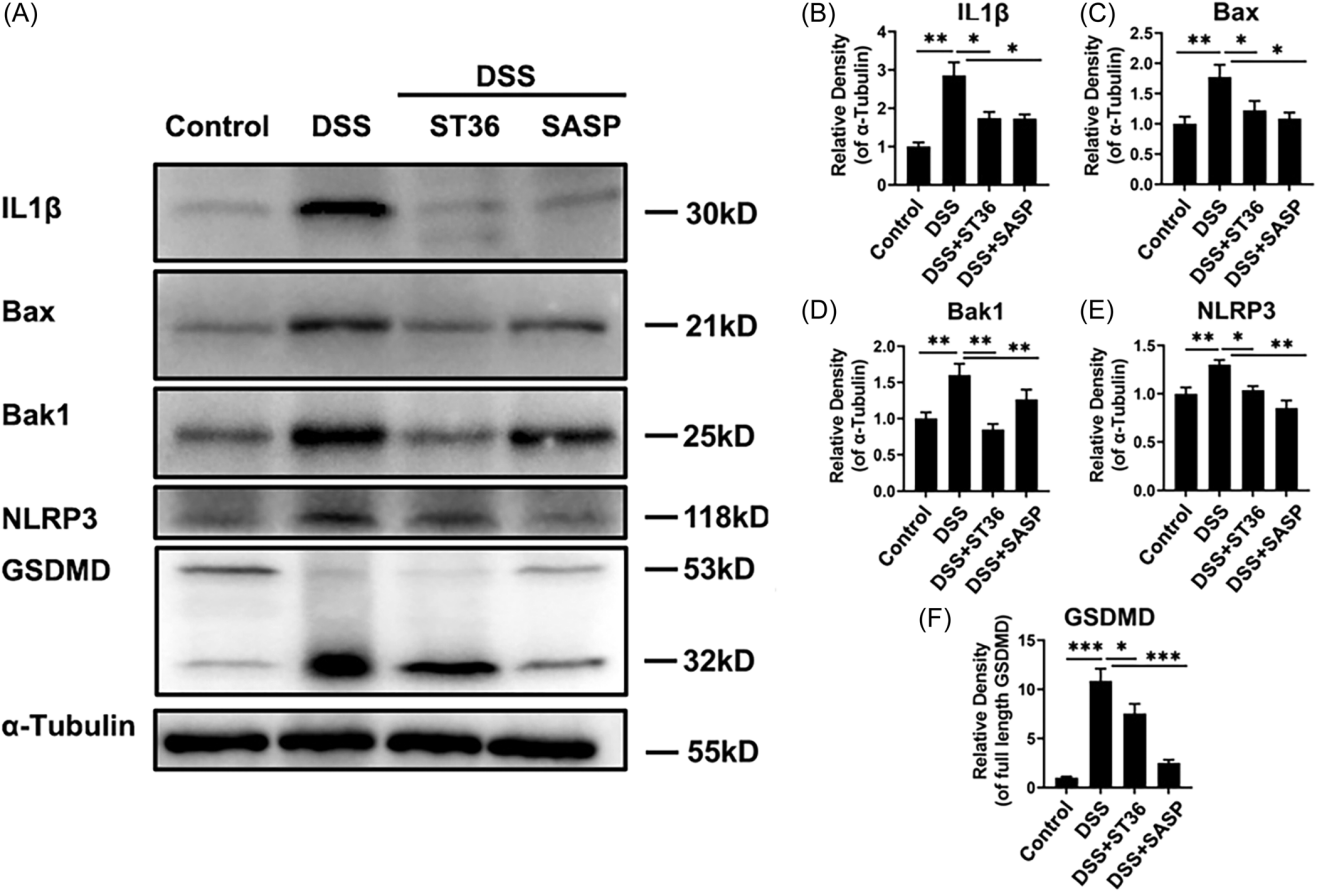
EA suppress the activation of NLRP3 inflammasome pathway in colon of IBD mice. (A−F) Protein levels of IL‐1β, Bax, Bak1, NLRP3, and GSDMD in IBD mice were determined using western blot analysis. The protein expression level was normalized against α‐Tubulin. The corresponding bands were quantified by ImageJ software. *p < .05, **p < .01, ***p < .001. EA, electroacupuncture; IBD, inflammatory bowel disease; SASP, sulfasalazine.

Accumulating evidence have suggested that low levels of NLRP3 reduce DSS‐colitis and TNBS‐colitis.^18^, ^19^ Herein, there were significant upregulation of NLRP3 pathways in DSS groups relative to the control groups as the evidence that DSS treatment enhanced the protein expression of Bax, Bak1, NLRP3, IL‐1β, and GSDMD‐N (Figure 6A−F) in the colon of IBD mice. Intriguingly, these increases of the above protein molecules in DSS groups were overturned after EA at ST36 and SASP treatments (Figure 6A−F). These findings conclude that EA stimulation at ST36 may attenuate the activation of NLRP3 inflammasome in the progression of IBD.

### 3.7 EA stimulation of ST36 affects intestinal tryptophan metabolism

**Figure 7:**
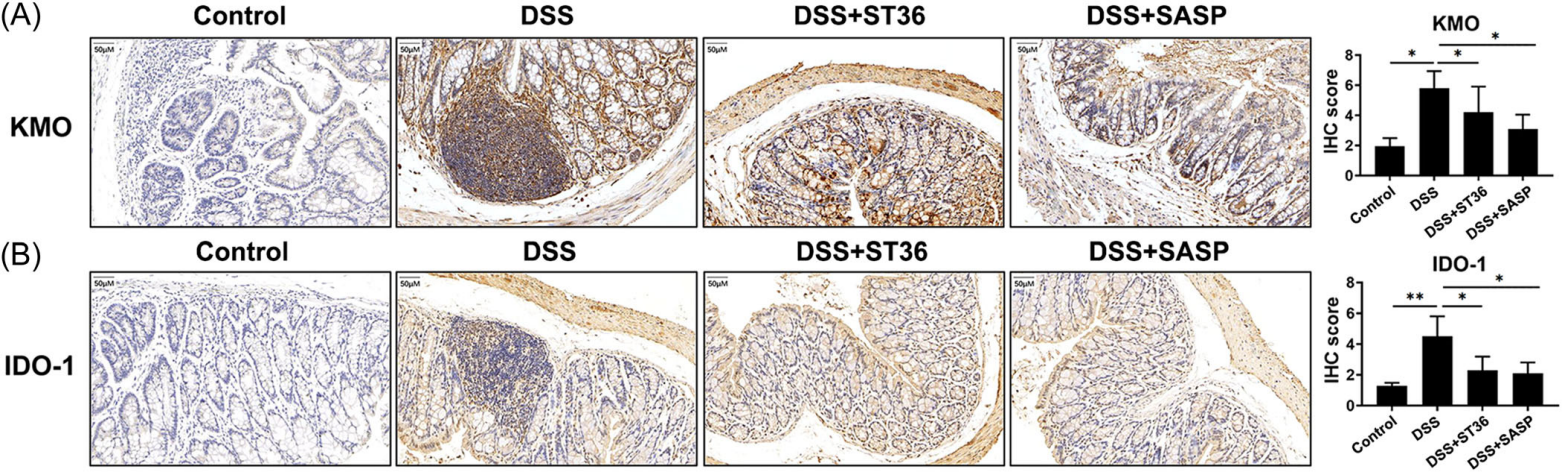
Immunohistochemistry (IHC) analysis of KMO, IDO in IBD mice. Mice were treated with DSS, EA at ST36 and SASP. Then, the KMO (A) and IDO‐1 (B) in IBD mice were evaluated by IHC. *p < .05, **p < .01. DSS, dextran sulfate sodium; EA, electroacupuncture; IBD, inflammatory bowel disease; KMO, kynurenine‐3‐monooxygenase; SASP, sulfasalazine.

Increased levels of Kyn, caused by regulating kynurenine‐3‐monooxygenase (KMO), may contribute to the induction of intestinal T cells and other immune cells during colitis.^36^ The activity of indoleamine‐2,3‐dioxygenase (IDO) may play a role in proinflammatory processes.^37^ This study showed that compared with the control groups, KMO (Figure 7A) and IDO‐1 (Figure 7B) were upregulated in DSS groups. However, stimulation at ST36 and SASP significantly reduced DSS‐induced elevation of KMO and IDO‐1.

## 4 DISCUSSION

**Figure 8:**
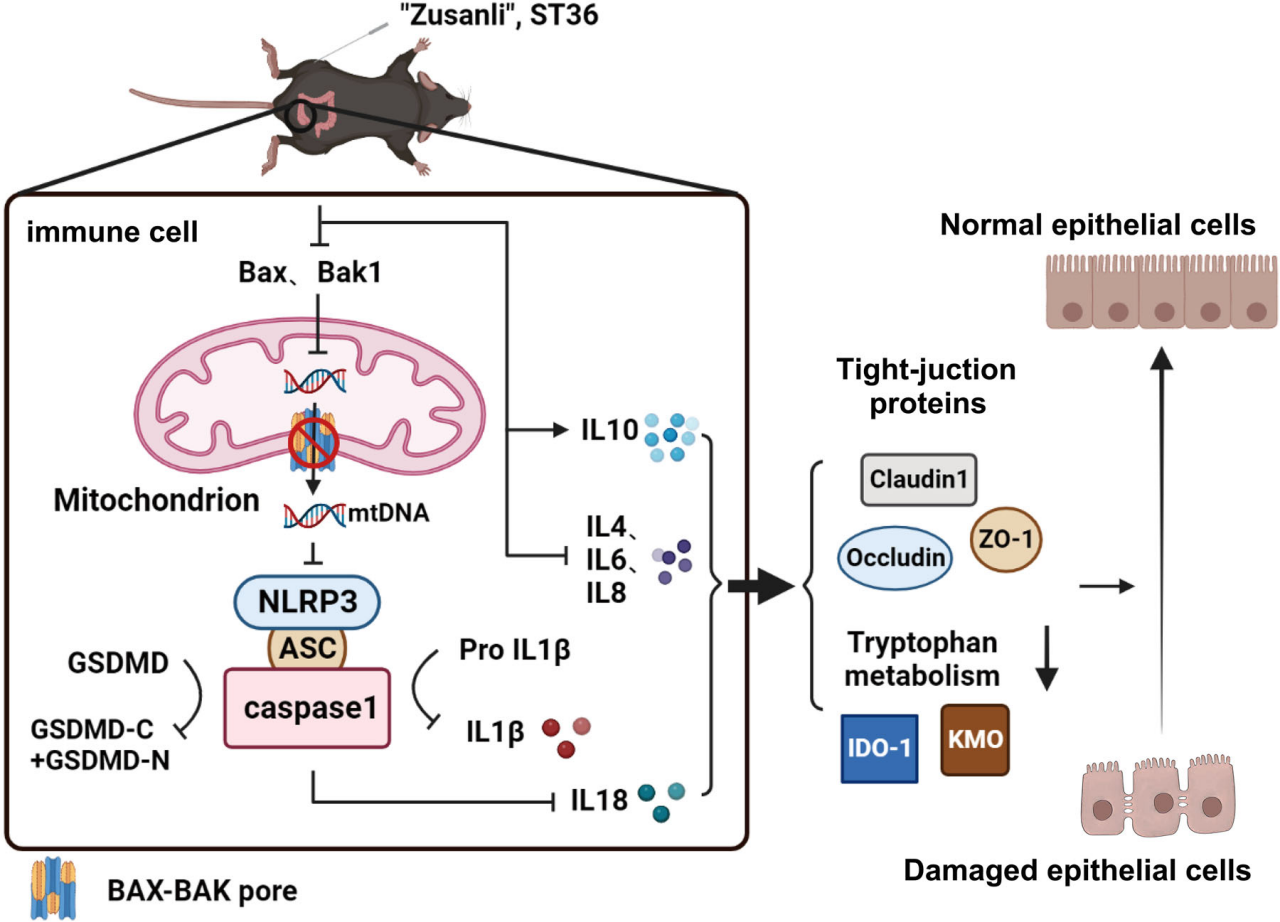
Schematics of electroacupuncture (EA) at “Zusanli” (ST36) effects on dextran sulfate sodium (DSS)‐induced acute colitis: EA at ST36 in IBD model decreases the expression of BAX and BAK1, inhibits the formation of BAX/BAK1 pore and prevents mtDNA leakage from colitis, which results in the inhibition of NLRP3 inflammasomes and abating the expression and splicing of GSDMD, IL‐1β, and IL‐18. Meanwhile, the release of proinflammatory factor IL‐4/IL‐6/IL‐18 and anti‐inflammatory factor IL‐10 were reversed after EA, which is responsible for the decreased cell death. Under the synergistic action of these two aspects, the increase of tight‐junction protein and the reduction of key proteins of tryptophan metabolism enhances proinflammatory processes in IBD, repair of damaged tissues, and attenuation of colitis. IBD, inflammatory bowel disease.

There is a plethora of research indicating acupuncture initiates a cascade of reactions that stimulates the production of neurotransmitters but is not exclusively focused on ST36. Acupuncture stimulation of ST36 activates the neurotransmitter network in the brain. In the brain, acupuncture stimulation at ST36 elicits widespread and synchronized signals in the cerebrocerebellar circuit; this is especially marked in the limbic system, which plays a central role in the regulation of immunological functions in IBD. Acupuncture triggers cross‐talk between the neurotransmitter network and the immune system.^38^ Herein, we focused on the effects of EA at the ST36 point in treating IBD (Figure 8).

NLRP3 inflammasome contributes to innate immune‐mediated inflammation and plays a crucial role in the pathogenesis of various autoinflammatory, metabolic, and neurodegenerative diseases. The main products of NLRP3 inflammasome are IL‐1β and IL‐18, which are involved in a variety of physiological activities. Increased IL‐18 expression contributes to the progression of TNBS‐induced colitis.^32^ IL‐1β is a common inflammatory cytokine and is involved in local and systemic responses to injury, infection, and inflammation.^20^, ^21^ Herein, Similar to previous results, our results confirmed the activation of NLRP3 and IL‐1β in the DSS group, and NLRP3 depletion after EA treatment resulted in reduced inflammasome aggregation and attenuated IL1β/GSDMD processing activities. The possibility that the NLRP3 inflammasome signaling pathway plays a key role in ST36's remission of acute colitis was again validated.

Recent progress in therapeutic approaches, especially the emergence of biologics, has promoted the transformation of the treatment mode in IBD and changed the perspective of IBD therapy.^7^ Amino acids are necessary for intestinal growth and maintaining the mucosal barrier. However, excessive amino acid intake can also produce harmful bacterial metabolites, which affect the repair of intestinal epithelial cells and damage them.^39^, ^40^ Previous studies have confirmed the changes in the levels of amino acid metabolites in patients during the pathogenesis of IBD.^41^ During the onset of IBD, intestinal cells release a large number of cytokines, which activate IDO‐1 and promote the activation of the tryptophan‐kynurenine metabolic pathway.^42^ Overproduction of kynurenine is associated with worsening of disease progression.^43^ Furthermore, studies have shown that intestinal tryptophan metabolism (kynurenine pathway [KP]) may play a role in proinflammatory processes in IBD.^37^, ^43^ Intestinal tryptophan metabolism genes are highly expressed in CD, UC, and other diseases.^2^ KMO is a key enzyme in the KP, and the activity of IDO, which catalyzes the rate‐controlling step in the KP, plays an important role in inflammatory diseases.^37^ Our findings revealed that KMO and IDO were significantly elevated in the sham group but were significantly downregulated in EA and SASP‐treated groups, suggesting that EA at the ST36 acupoint suppressed intestinal tryptophan metabolism (KP). Additionally, this research aims to elucidate the interrelationship between the NLRP3 and intestinal tryptophan metabolism in the framework of EA stimulation therapy. Consequently, our future research plan entails a more detailed investigation into the dysregulation of intestinal tryptophan metabolism mediated by KMO and IDO in the context of IBD.

## 5 CONCLUSION

EA on the Zusanli acupoint improves IBD symptoms, providing new mechanism for clinical interpretation in IBD. EA at Zusanli improves IBD by manipulating the NLRP3 and IL‐1β/IL‐18, proposing a new mechanism of EA acting on the intestine (Figure 8). Our findings exhibit that EA at Zusanli regulates the intestinal tryptophan metabolism which can manipulate the IBD pathogenesis that may play a significant role in clinical research of IBD. This study may unveil new therapeutic targets for the clinical elucidation of IBD.

## AUTHOR CONTRIBUTIONS

**Miaomiao Cai**: Data curation; investigation; methodology; resources; software; supervision; validation; visualization; roles/writing—original draft. **Yanqiang Chen**: Data curation; investigation; methodology; formal analysis; data curation; resources; software; supervision; validation; writing—review and editing. **Boyuan Shen**: Data curation; methodology; investigation; formal analysis; data curation; validation; resources. **Changchang Fan**: Data curation; software; validation; methodology; formal analysis; software; validation; formal analysis. **Xiang Zhou**: Conceptualization; data curation; formal analysis; investigation; methodology; project administration; software; validation. All authors participated in the design, interpretation of the studies and analysis of the data and review of the manuscript.

## CONFLICT OF INTEREST STATEMENT

The authors declare no conflict of interest.

## ETHICS STATEMENT

The Animal Ethics Committee of Wuhan University of Science and Technology approved all experimental animal experimental protocols and complied with the National Institutes of Health Guidelines for the Use and Care of Laboratory Animals (published by the National Institutes of Health, Edition: 85‐23, revised in 1996)

## Data Availability

The data sets used and/or analyzed during the present study are available from the corresponding author upon reasonable request.
